# Editorial: Model-informed approaches: uniting drug development with personalized medicine

**DOI:** 10.3389/fphar.2026.1899395

**Published:** 2026-06-23

**Authors:** Francine Johansson Azeredo, Venkata Kashyap Yellepeddi, Bibiana Verlindo de Araujo

**Affiliations:** 1 Center for Pharmacometrics and Systems Pharmacology, College of Pharmacy, University of Florida, Orlando, FL, United States; 2 Division of Clinical Pharmacology, Department of Pediatrics, University of Utah School of Medicine, Salt Lake City, UT, United States; 3 Department of Molecular Pharmaceutics, College of Pharmacy, University of Utah, Salt Lake City, UT, United States; 4 Programa de Pós-Graduação em Ciências Farmacêuticas, Universidade Federal do Rio Grande do Sul, Porto Alegre, Rio Grande do Sul, Brazil

**Keywords:** drug development, model-informed drug development (MIDD), personalized medicine, pharmacometrics, PK/PD model

The increasing complexity of drug development and the growing demand for individualized therapies have accelerated the adoption of model-informed approaches across translational and clinical pharmacology (Cheng et al., 2025; Kapitanov et al., 2026). Quantitative frameworks integrating pharmacokinetics, pharmacodynamics, systems pharmacology, machine learning, and real-world clinical data, transforming how therapies are designed, optimized, and implemented in clinical practice (Lin et al., 2020; Mehta et al., 2024). This Research Topic, *Model-Informed Approaches: Uniting Drug Development with Personalized Medicine*, highlights recent advances demonstrating how computational and mechanistic strategies can bridge drug development and precision medicine.

The articles assembled in this Research Topic illustrate the broad applicability of model-informed methodologies across therapeutic areas, patient populations, and stages of drug development. Together, these contributions emphasize the growing role of pharmacometrics, physiologically based pharmacokinetic (PBPK) modeling, quantitative systems pharmacology (QSP), machine learning, and digital health technologies in supporting evidence-based therapeutic decisions.

Most studies have focused on improving dose optimization in vulnerable populations through mechanistic modeling. Feng et al. developed a PBPK model to characterize imipenem pharmacokinetics in pediatric patients with renal impairment, providing a framework for individualized dose adjustment in a population with highly variable physiology. Similarly, Gao et al. systematically evaluated published population pharmacokinetic models of mycophenolate sodium and externally validated these models in Chinese renal transplant recipients, highlighting the importance of model qualification and extrapolation across diverse patient populations.

Precision medicine strategies integrating computational modeling with clinical biomarkers and patient-specific characteristics were also strongly represented. Zhou et al. demonstrated that central nervous system exposure and analgesic response to gabapentin are influenced by OCT2 genotype in patients with chronic neuropathic pain, reinforcing the clinical relevance of pharmacogenomics-informed dosing strategies. Chen et al. applied machine learning-based covariate ensemble modeling to optimize infliximab concentration monitoring and dose adjustment in Crohn’s disease patients, illustrating the expanding role of artificial intelligence and data-driven methods in therapeutic individualization.

The issue additionally highlights emerging systems-level and translational methodologies. Hu et al. combined quantitative systems pharmacology modeling with patient-derived organoid testing to investigate treatment strategies in MET-aberrant non-small cell lung cancer. This innovative integration of mechanistic modeling and experimental platforms represents a promising direction for personalized oncology and high-throughput therapeutic evaluation. Expanding beyond pharmacology alone, Garbey et al. constructed patient trajectories to model clinical trial outcomes in myasthenia gravis, demonstrating the utility of integrating longitudinal data and modeling disease progression in clinical research.

Importantly, this Research Topic also addresses the future landscape of model-informed medicine. Kimko et al. presented a perspective on the application of digital twin technologies to transform prescription drug labeling, proposing a framework in which individualized virtual representations of patients may support safer, more precise therapeutic recommendations. Such approaches exemplify the convergence of computational sciences, clinical pharmacology, and digital medicine.

Collectively, the studies presented in this Research Topic reinforce the notion that model-informed approaches are no longer limited to supportive tools in drug development but are increasingly central components of therapeutic decision-making and precision medicine. The future of the field will likely be shaped by integrating multimodal clinical data, artificial intelligence, real-world evidence, and mechanistic modeling into unified frameworks that support individualized treatment strategies in real time (Chu et al., 2025; Mannix et al., 2026). Advances in computational power, digital health technologies, and systems-based methodologies are expected to further accelerate the development of patient-centered therapeutic approaches, enabling more predictive, adaptive, and efficient healthcare (Rudrapatna et al., 2025). As these methodologies continue to mature, model-informed strategies will play an increasingly important role in bridging translational science and clinical implementation, ultimately contributing to safer, more effective, and more equitable therapeutic interventions.
